# Psychological Impact of Hospital Discharge on the Older Person: A Systematic Review

**DOI:** 10.3390/geriatrics9060167

**Published:** 2024-12-20

**Authors:** Yasmin Hussein, Sarah Edwards, Harnish P Patel

**Affiliations:** 1 Medicine for Older People, University Hospital Southampton NHS Foundation Trust, Southampton SO16 6YD, UK; 2 Academic Geriatric Medicine, University of Southampton, Southampton SO16 6YD, UK; 3 NIHR Biomedical Research Centre, University Hospital Southampton, Southampton SO16 6YD, UK

**Keywords:** older adult, post discharge, psychological vulnerability, morbidity, mortality

## Abstract

**Introduction:** Hospitalisation and prolonged length of stay is associated with deconditioning that risks adverse outcomes after discharge. Less is known about the psychological impact on older people after hospital discharge. The purpose of this systematic review was to elucidate factors contributing to psychological stress in older patients post-discharge to inform better discharge planning. **Methods:** A systematic search for studies reporting poor discharge outcomes in older people between 2010 and 2022 was performed in Medline, CINAHL, and PsycINFO. Search terms were ‘older patients > 65 year’, ‘post-discharge’, ‘psychological distress’, ‘loneliness’, ‘anxiety’, ‘depression’, and ‘length of hospital stay’. Exclusion criteria included COVID-19 disease, dementia (±severe cognitive impairment), individuals aged <65, and those under palliative care services. **Results:** A total of 1666 records were identified, of which 878 were excluded as they were outside of our date limits or were not written in the English language, 681 were excluded after application of exclusion criteria, and 699 were excluded because of insufficient details. A total of 31 duplicates were removed, leaving 38 articles that were assessed for eligibility; 7 of these reports were found suitable, comprising 1131 patients. Three highly relevant themes identified relating to post-discharge outcomes were social isolation, lack of support, depression and anxiety. Older patients with a tendency toward depressive symptoms had an increased likelihood of death. **Conclusions:** It appears that the discharge process from hospital fails to address psychological factors that permit a successful transition from hospital. Pre-discharge screening of psychological symptoms and coping ability may assist in identifying older patients who are at risk of mental as well as subsequent physical deterioration. Better knowledge of positive and negative predictors of a successful transition from hospital to home would enable more holistic, effective, and inclusive discharge planning processes for older adults.

## 1. Introduction

Approximately 19% of the population is accounted for by individuals who are aged > 65 years. This proportion is due to increase to 22% by the year 2030 [1]. Therefore, older people represent the fastest-growing section of the population. Whilst these demographic changes worldwide and in the UK are a testament to advances in medical care allowing individuals to live well with one or more long-term conditions and frailty, the provision of integrated health and social care remains a challenge. For some older adults, decompensation in their health status necessitates unplanned hospitalisation, which is appropriate for the treatment and management of the index reason for admission. However, admission to hospital involves a sequence of events culminating in multiple healthcare contacts where older adults not only have to contend with their symptoms, pain, fear, and undernutrition, but also assimilate complex information about diagnosis, treatment and investigations that may not be accurate or may change day by day. Deleterious effects on health spanning across one or more physical or psychological domains include the risk of nosocomial infections [2]; medication interactions and medication-related harm by virtue of in-hospital prescribing in addition to existing polypharmacy [3]; delirium [4]; medical and surgical complications [5]; functional decline [6]; as well as loss of autonomy, decreased confidence and dignity, increased dependence, stress, and uncertainty of whether they will ever get better [7,8]. Recent research has shown that patients may also be at risk for cognitive decline after hospitalisation [9]. Previous data has shown that an average of 35% of older individuals do not recover functionally at the time of hospital discharge, regardless of the population evaluated and the place of hospitalisation [10]. Older adults hospitalised for serious injuries due to a fall are exposed to a significant risk of adverse events post-discharge, such as a new fall, functional decline, and emergency visits. These patients are more likely to be readmitted for a fall within 30 days of discharge [11,12,13].

Multiple studies have reported that older patients experience a discontinuity of care on the journey back into their community [7]. This allows vital information, such as one’s individual worries and emotional state, to be missed in the discharge process. For example, if an older person at the time of hospital discharge shares reasons to believe that they will have difficulties with psychological distress at home, this information may not be divulged to the appropriate staff member due to a possible lack of questioning. The reasons are multifaceted but when considering the extremely busy and fast-paced nature of hospital wards, the subtle nuances in a patient who is worried about discharge can be missed. In support of this, Bull et al. [14] found that the best predictors of older people’s satisfaction with discharge planning were a perception of continuity and care and how well-prepared the patient felt by the hospital teams. Hoping to address the problem in care transitions and to reduce the gaps in service delivery, different intervention models have been implemented. These include post-discharge planning in hospitals and the provision of post-acute care [15]. Many of these models, however, focus on the medical needs of patients and pay notably lesser attention to the individual psychosocial needs of patients in the community which, while unrelated to their medical presenting complaint, nevertheless affect their post-discharge recovery.

Although the relationship between hospital stay and functional decline is evident worldwide, less is known about the relationship between psychological risk factors and how patients cope with the demands of life post-discharge. The purpose of this systematic review was to elucidate factors contributing to psychological stress in older patients post-discharge. A better understanding of the inter-individual determinants that predict resilience levels and coping mechanisms in older people will enable more efficient and holistic discharge processes.

## 2. Methods

The protocol for this systematic review has been written in accordance with the Preferred Reporting Items for Systematic Review (PRISMA) guidelines [16]. Research studies with clearly stated hypotheses were included in the study. Secondary literature such as systematic reviews, meta-analyses, and other systematic meta-reviews were excluded from the review as this was an exploratory investigation into the effects of hospital discharge on the psychological state of patients. However, some of this literature was used as scoping material prior to the formal application of the methodology process. At this stage, Google Books was also used as a source of scoping material. Broad questions were searched via Google such as “loneliness in older patients”, “association between poor mental health and poor adherence to treatment”, and “the impact of having many healthcare professionals caring for a single patient”. None of these knowledge streams were to be used in the final review, but they served to expand subject knowledge.

The population of interest for this review was older patients > 65 years while considering that a strict definition for “older” is difficult due to the differences in the rate of biological ageing between individuals (>65 years of age was used to help standardise the search). The chosen research would follow the aim and include patient accounts post-discharge as well as identify the presence of *any* intervention used pre-discharge within the context of the research question.

A comprehensive literature review using Medline, CINAHL, and PsychINFO was conducted. The search was limited to studies published in the English language between 2010 and 2022 which aimed to recognise findings that reflect current experiences. The selection of articles to be included was carried out by a single reviewer following a three-step process: (1) title screening; (2) abstract screening, and (3) full-text screening. Documents were found eligible if they included a population of interest of older patients (>65 years) navigating the transition from hospital to home as well as an account of patients’ experiences. Additionally, articles were eligible if they included a concise review of *all* older people without differentiating between sexes. Evidence reported in all articles focused solely on the viewpoints of the >65-year-old population, driving a complete review of both sexes. The objective of the articles was to concisely report the lives of older patients after hospital. However, it was not required that all articles include a review of patient’s lives prior to hospitalisation. The timescale for a review of a patient’s transition from hospital to home environment was not scrutinised in the chosen articles. See Appendix A Table A1 for the search terms used in Medline, PsycINFO, and CINAHL.

### 2.1. Inclusion Criteria

“Aged > 65, post-discharge and psychological distress” were thought to best represent the target population needed for the review. As opposed to ‘pre-discharge’, post-discharge reflected a point in time that this review aimed to explore. Further subject headings added were “loneliness”, “anxiety”, “depression”, and “length of hospital stay”. Abstracts for all papers were reviewed following results from the search terms to include the specific points of the inclusion criteria. The inclusion criteria were listed from highest to lowest priority to best reflect the situation from an epoch in the older person’s life (Table 1).

### 2.2. Exclusion Criteria

Papers that included age groups < 65 years were removed from the search immediately following a review of their contents. This was to select papers that included rich data on the target group needed for this review. Pre-discharge was used as an exclusion point only if papers included this period solely and not in conjunction with the post-discharge period. In addition, studies that included patients living with dementia/severe cognitive impairment, or who were under palliative care were removed, negating the influence of cognitive disorders on the psychological state of the older person, and the fact that patients on the palliative care pathway have predicted trajectories and follow-up. Studies exploring the effects of the COVID-19 pandemic were removed from the review (Table 1).

## 3. Results

**Figure 1:**
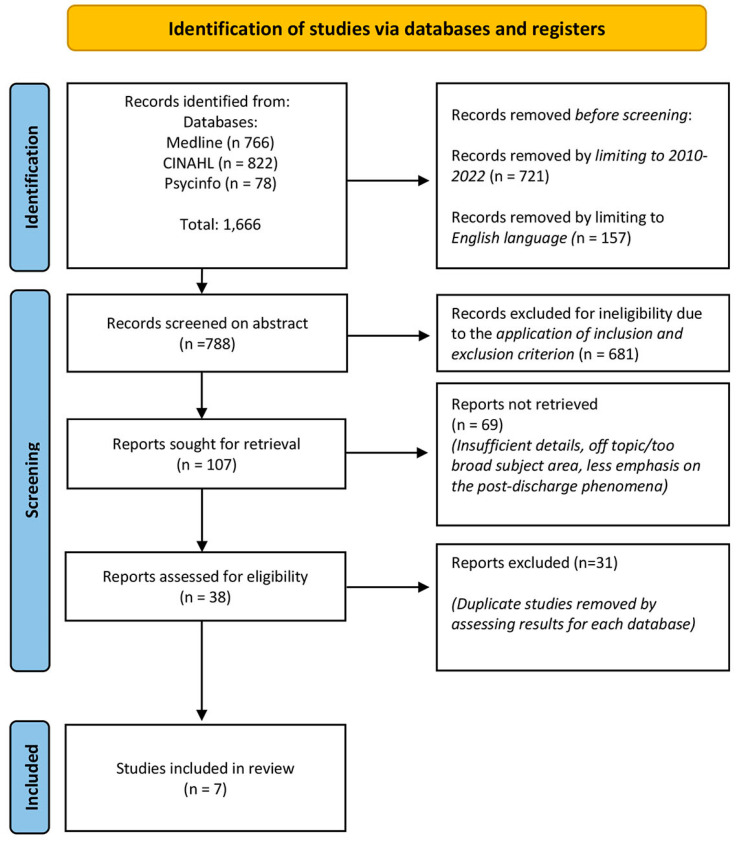
PRISMA flow diagram for included studies.

Searches on electronic databases initially produced 1666 citations for eligibility screening. From these, 878 were removed after confining to English language publications ranging from 2010 to 2022. Upon abstract review, a further 69 papers were removed due to insufficient data on the post-discharge phenomena. The latter resulted in 38 papers, which once screened for duplicates, left 7 eligible papers to be used for review (Figure 1).

### 3.1. Included Studies

Following from identification of total papers and screening the data, a total of seven studies (with 1066 participants) from five countries met the inclusion criteria (Table 2). They were conducted in the following countries: the USA [17], Denmark [18,19], Australia [20,21], Sweden [22], and the Netherlands [23] (Table 2). The study types were interpretive, prospective, descriptive, and phenomenological. The included studies were conducted between 2014 and 2020. All studies included in this review included participants recruited from a hospital environment or intermediate care. Each of the studies explored the experiential nature of the lives of older patients post-discharge, resulting in a predominantly qualitative data set with the use of interviews, surveys, and rating scales. However, three studies [20,21,23] did include quantitative data and used logistic regression analyses to derive odds ratios (Table 3).

### 3.2. Synthesis of Findings

Information was extracted from the data sources (e.g., year of publication, authors, study types), including the characteristics of the population (e.g., gender, age). The retrieved papers were assessed by two authors independently, reducing the chance of reporting bias. The aims, study design, data analysis methods, and summary of findings were collated into Table 3. A narrative synthesis of the findings was performed. Key ideas from retrieved articles generated a set of codes that were then categorised into themes that were agreed upon by two authors highlighting older adults’ experiences of hospitalisation, discharge, and post-hospital transition. These themes were (1) social isolation; (2) issues with support from the system; and (3) depression and anxiety.

### 3.3. Analysis and Summary of Findings

#### 3.3.1. Theme 1: Social Isolation

Most participants in the included papers experienced a transition to home coupled with a loss in social life. Regardless of their health presentation, this negatively impacted their recovery. Participants unanimously agreed that social support was a crucial component of recovery from hospitalisation. Limitations to social activities were felt almost immediately by patients due to unanticipated decreases in functional ability after discharge. One 63-year-old woman expressed during her interview that it was as if she was “in her own little world” following returning to the community [17]. Older participants felt as though the social dimension of their existence was the most crucial component of their everyday lives. Losing autonomy in social life meant that these patients were more likely to be secluded from the community, and with loss of contact came great distress [18]. The unique determinants of everyday mood were predominantly emphasised in the study by Andreasen J et al. [18]. This study found that the more challenges the older person experienced in their everyday life, the more difficult it was to maintain a positive mindset. The influence of mindset revealed the different ways in which older people may individually deal with their lives after discharge. For example, it was found that two older female participants presenting with the exact same medical conditions, one living in a house with her husband and the second living alone in a third-floor apartment for a total of three decades, had contrasting home experiences post-discharge. The former participant experienced no degrees of physical disability post-discharge aside from tiredness, whereas the latter suffered back pain, no appetite, and feelings of weakness. When this participant was asked by researchers what she most valued, her response was “nothing” due to there being “no meaning to anything”. The same study emphasised how the lack of participation in meaningful activities caused older people to feel alone after discharge, and some expressed a wish to die.

Counter to this, acceptance of the ageing process meant that some participants were able to accept the deterioration of physical function as part of a natural process [22]. Patients felt that they needed to change something about their lives after hospitalisation whilst also becoming reliant on using the television as a replacement to maintain some aspect of social contact [18,22]. The importance of the outdoors was emphasised by the participants as interaction with neighbours, family, and friends increased the informants’ perceived “quality of life” and independence. Experiencing “shock” was explored in the study by Martinsen et al. [19], which highlighted the reality of the transition an older person living with frailty faced after discharge from an intermediate care (IC) unit. Regardless of how prepared the discharge was by staff, these patients felt considerable setbacks in recovery due to formal rehabilitation classes being either stopped or drastically reduced. This meant that managing daily activities such as personal hygiene demanded a great deal of strength and thus left less room for social activities. These patients had access to help from caregivers and interaction with other patients during their stay in the IC unit. This experience further aggravated the experience of isolation coupled with bodily limitations post-discharge [19].

#### 3.3.2. Theme 2: Issues with Support from the System and the Community

The patients felt that the system provided for them but not in relation to their own perceived and expressed needs. Some participants felt as if their needs were not met after discharge and feelings of being objectivised were shared, leading to heightened frustration. For example, predetermined home visits by the welfare team without considering the personal needs and wants of the patient led to frustration. One patient shared that the tasks she needed help with the most were not met by the provision of a standard package of care. Another patient in the same cohort who lived on a farm felt “violated”, perceiving he was battling the system because his location precluded the provision of care, and was heavily reliant on his wife to assist him in his recovery. This led to feelings of aloneness or abandonment in both [18]. Many patients also elaborated on specific issues that had not been addressed in the discharge process, stating they did not feel the discharge plan encompassed how they would return to their normal everyday activities.

Relatives, mainly adult children, played a significant role when the older people returned home such as meeting them at the hospital unit and taking them home. However, there was an awareness that they should not burden their family with providing care. This meant the older person reached out to carers in the public system, which was often met with criticism due to the rigid nature of home visit appointments [19]. Patients who had family close by also felt they were not able to rely on them for support during the recovery period post-discharge. Some reported difficulties with transportation and coordinating schedules as major obstacles to receiving help from family members, with one patient expressing that her daughter “doesn’t come when she’s supposed to” and that it makes her “depressed” [17]. Another informant mentioned that she asked her children for help, although she preferred turning to a friend her own age for emotional support. Patients without informal support and with first-time home-help service found it intrusive as they did not have “control over their own day”. Patients also felt the peculiarity of having each of their tasks planned, such as when to take a shower, added an air of abnormality to their new reality [22].

#### 3.3.3. Theme 3: Depression and Anxiety

After hospitalisation (within 48 h from admission) with the use of the Geriatric Depression Scale (GDS-15), symptoms of apathy were the most reported depressive symptom. More than 70% of a cohort of 398 older people reported a loss of energy during hospitalisation, with more than 50% preferring to be at home, and 45% giving up on their activities and interests. The total score on the GDS-15 was found to be a significant predictor of mortality when measured within 48 h after admission (OR 1.2, 95% CI 1.1, 1.3) and at discharge (OR 1.2, 95% CI 1.1, 1.4). The strongest associations between the depressive item collected within 48 h after admission and mortality within 3 months post-discharge were found for two items: hopelessness (item 14, ‘Feelings of hopelessness about their situation’: (OR 3.6, 95% CI 1.8, 7.4) and item 8, ‘Often feeling hopeless’: (OR, 3.4, 95% CI 1.6, 7.3). More than 40% of the patients who reported feeling hopeless about their situation at admission died between admission and 3 months post-discharge. When measured at discharge, this increased to 45% [23].

Furthermore, the use of the Social Antecedent Model of Psychopathology (SAMP) stages in the study by Brown et al. [20] indicated that depression was higher in males, those with greater anxiety symptoms, low social support, and lower perceived coping ability. Baseline depressive symptoms (*β* = 0.09, *p* = 0.01) were significantly associated with future depressive symptoms at 3 months post-discharge. As well as this, when controlling for baseline depressive symptoms, lower baseline household physical activity levels (*β* = 0.02, *p* = 0.02) were associated significantly with symptoms of depression 3 months post-discharge. Furthermore, baseline depressive symptoms (*β* = 0.06, *p* < 0.001) were significantly associated with future depressive symptoms after 6 months, along with higher baseline anxiety symptoms (*β* = 0.02, *p* = 0.02) and lower social support (*β =* 0.02, *p* = 0.03). Additionally, in the study by Lee et al. [21], it was found that participants who had one or more falls during a particular month had higher scores for depression at the same assessment point. A higher score for depression was also associated with an increased likelihood of reporting one or more falls in follow-up assessments. Finally, reporting higher levels of depression was significantly associated with lower levels of household (unadjusted coefficient: −0.07 (−0.09, −0.05), *p* < 0.001) and recreational physical activity levels (unadjusted coefficient: −0.04 (−0.05, −0.02), *p* < 0.001). This combination was significantly associated with one or more falls one-month post-assessment.

## 4. Discussion

This systematic review synthesized evidence concerning the experiences of older adults after hospital discharge. The aim of the study was to elucidate factors contributing to psychological stress in older patients post-discharge, which may inform more efficient and holistic discharge planning processes.

We have found that older adults experience a discontinuity of care on their way from hospital back into their community which is also supported by the existing literature [7]. Older participants in our review felt as though the social aspects of their lives were most crucial, and since many lived alone, i.e., after recently being widowed, they represent a highly vulnerable group. A 2011 study by Newson et al. [24] found a higher prevalence of complicated grief in people aged 75–85 years, suggesting that older people have more difficulty in coping with loss. This feeling of loneliness has dire consequences on the degree of fulfilment of the older person. High-quality social connections are a prerequisite for mental and physical health at all ages. Studies have shown that individuals with the lowest levels of involvement in social relations were more likely to die than those with greater levels of involvement. Our review also found that illness may go unnoticed due to social isolation. This means that many older individuals in society could find themselves in debilitating situations but are unable to seek help as they are away from the view of the public and healthcare services [25].

Loneliness can be defined as the perception of social isolation or the subjective feeling of being lonely. Loneliness can be used synonymously with isolation, abandonment, rejection, sadness, aloneness, withdrawal, and seclusion. Social isolation and loneliness among older adults have become major concerns. Age UK, a large nonprofit charity for older people in the United Kingdom, reported that loneliness has been “*…proven to be worse for health than smoking 15 cigarettes a day*”. To support this claim, findings from a meta-analysis indicate health, e.g., immune functioning and cardiovascular decline, was associated with the quality and quantity of social relationships, as well as a higher mortality risk in those who are more isolated [26]. The analysis touched on previous work that highlighted a mortality risk 1.50 times higher in those who were lonely, similar to the risk from light smoking, and exceeding the risks conferred by hypertension and obesity. Thus, the epidemiological data suggests that having better quality relationships is linked to decreased health risks. Having fewer and poorer quality relationships increases this risk [27]. Another study conducted by researchers from the University of California found that adults >60 years old who reported feeling lonely were at a higher risk for functional decline in addition to the increased risk of death [28]. This decline manifested specifically in the ability of participants to perform activities of daily living (ADLs) such as bathing, dressing, toileting, continence, and eating, which are necessary for independent living after discharge from hospital. Unchecked, loneliness can hinder an individual’s functional ability, accelerating the need for third-party assistance and potentially resulting in referral for long-term social care.

A 2017 report from the Silver Line Charity, a UK-based 24 h a day, 7-day-a-week confidential telephone service, found that increasing numbers of older people with issues such as depression, loneliness, suicidal thoughts, and fear of death were calling for companionship. The report continues to explain that many older patients refrain from speaking to family due to fear of losing their independence. Furthermore, when considering the impact of the 2020 COVID-19 pandemic, many of these individuals may keep themselves away from caregivers due to (1) fear of being a burden, and (2) fear of progressive health deficits. In support of this, studies have reported an increase in anxiety and depression in the general population that was magnified in the older population due to stricter lockdowns, increased threats from illness, and a loss of social support [29,30]. This may be due to emotional distress precipitated by the absence of social contacts and participation in self-medicating activities within their own homes without external oversight from social or healthcare services [31]. To combat social isolation, communities must actively incorporate the older population into every aspect of society regardless of attending age groups. This could be achieved by arranging bi-weekly activities at home or at day centres as well as notifying individuals of local social activities. Governments, having highlighted the impact of loneliness on the older population, and in partnership with local councils, must work with communities to build a bridge between helpers and those who need to be helped. Additionally, this review revealed the polarising effects of mood on the degrees of capability in the older population [18]. Studies have found that emotional well-being is a strong predictor of long-term prognosis of physical illness, suggesting that enhancing well-being could improve the prognosis of physical illness [32]. Therefore, strategies are required to uplift health and well-being through access to coping mechanisms created by other like-minded people, i.e., other older people.

A further theme highlighted by this review was around support from the system and community, and revealed older adults felt their physical needs such as medication requirements were being met yet personal and psychological requirements were not being fulfilled. A scoping review of the care and support needs of older people highlighted that many older adults wanted to stay at home and manage their illness, but hindrances to this included a lack of professional advice on self-care strategies, poor communication and coordination of services, and a lack of information on services such as care pathways/whom to call in times of heightened health anxiety [33]. In addition, our review also found problems with self-care and completing ADLs. Guilt over reliance on informal care from their own families was a worry for older adults, suggesting they required care and support with maintaining social activities and relationships, and help with psychological health and activities associated with mobility, self-care, and domestic life [33]. The literature also suggests patients and both formal and informal caregivers were often unclear about who was responsible for coordinating care needs and which health service to contact for advice. Thus, older adults often struggled to manage their condition(s) and symptoms after discharge and felt frustrated by not receiving clear directions on management. [34].

One finding in this review related to the impact of depression and anxiety on the overall livelihood and longevity of life of the older population. Older patients who had experienced higher levels of anxiety in hospital went on to have even higher levels once back in the community. This indicates that the observed increase in anxiety is causally related to a change of location and is not due to a further decline in their health. Depression at baseline was associated with mortality up to 3 months and 6 months post-discharge in the older population. Additionally, upwards of 40% of patients who reported hopelessness had died between admission and 3 months post-discharge. Psychoneuroimmunology is a novel aspect of medicine exploring the impact of psychological factors on physical health. Studies have shown that physiological ageing can modify one’s responsiveness to stress because of reduced resilience [35]. This adaptive physiological response to stress was initially referred to as allostasis by Sterling and Eyer in which the internal environment fails to meet the demands of the external world [35]. Studies have shown depression to be associated with a shortened life expectancy [36], with estimates of a 50% increased risk of mortality [37]. Estimates also suggest that over half of people with major depression had their first onset in old age [38]. Furthermore, it was found that older adults with late-life depression used more medical services, with total healthcare costs estimated to be 47% to 51% higher for patients with depression as compared to those without [39]. Older adults facing depression may lack the motivation to assist in self-care regimes such as taking prescribed medications on time, adequately nourishing themselves, and attending follow-up appointments to assess disease progression. In support of this, studies have found that older people with high levels of depressive symptoms have poorer treatment adherence, longer hospital stays, increased hospital readmission, and reduced functional status [40]. The prevalence of depressive symptoms can be as high as 50% among hospitalised older adults, highlighting the significance of hospital stay on the mental health of patients [41].

However, although depression is a common condition, many patients may be left untreated. As with anxiety disorders, the difficulty lies in differentiating between an acute episode of psychological distress and a chronic episode in the hospital environment. Hospital staff may innocently assume the cause of an older patient’s low mood to be a result of their physical health, meaning these vulnerable older patients return to their homes with an even larger psychological load to bear. This load is both detrimental to recovery from acute illness and negatively impacts their physical capability. A better understanding of the factors associated with high depressive symptoms post-discharge may provide information that is useful in (1) identifying those who are vulnerable to post-discharge depression and (2) actively assessing and treating risk factors during and after hospitalisation. Multimorbidity is commonly defined by the presence of two or more long-term conditions, which can be managed through medications, lifestyle changes, or other treatments [42]. By the age of 65 years, 75% of individuals will have a least one chronic disorder and by the age of 85 years, 95% of individuals will have at least one comorbid illness [43]. Previous research has indicated the existence of co-morbid mental health issues alongside long-term physical health conditions is particularly pernicious to well-being. A negative spiral between an already mentally vulnerable individual and the presence of comorbid illness predisposes an individual to an increased risk of mortality. Recently, an increasing number of studies have focused on the effect of multimorbidity on psychological distress. One study found that the prevalence of mental illness increases with the number of chronic conditions, suggesting that living with a multimorbidity contributes to deficits in psychological well-being and not merely an individual’s physical well-being [44]. Additionally, chronic physical conditions and symptoms are consistently associated with an increased tendency towards depressive symptoms. Fatigue and impaired physical functioning can lead to social isolation. In an already demanding life situation, those with depression and anxiety who are dealing with multimorbidity would likely find it harder to cope with the everyday struggle of their condition(s). In another study, it was found that patients described the period post-discharge as not being centred around their needs but based on the structure of the healthcare system. This suggests insufficient information on the diagnosis and treatment at the point of hospital discharge was being shared with the patient, adding further stress to an already psychologically vulnerable patient [45].

### Limitations and Strengths

We applied predefined inclusion and exclusion criteria to narrow the focus of our search commensurate with the research question and reduce bias in the selection of studies. Each study was critically reviewed for quality and relevance. We are very aware that there are numerous well-documented problems faced by patients post-discharge, such as physical deconditioning, medication errors, and poor communication amongst healthcare professionals, that impact the safe transition from hospital to home [7,34,46,47]. In addition, this systematic review was limited in the sense that all studies took place in Western countries. This left more room for bias when evaluating the true lived experience of older adults. What may be considered acceptable for individuals in the West may not be for those in the East. From our study, there was a realisation that older people hesitate to reach out for help from younger populations. However, we did not explore why this was the case. A future study may be able to break down the resistances and obstacles that younger and older individuals have that preclude both giving and asking/receiving help, respectively. These studies may then identify tangible interventions that can maintain the independence, health, and well-being of older people. Other limitations were that main health databases were employed so some publications may have been missed; ethnic minorities may not have been captured within the retrieved articles; and the use of studies published in English, as well as the absence of grey literature, may have introduced publication bias. Nevertheless, this study has several strengths. We have identified key factors that appear to be important and must be considered and accounted for when planning hospital discharges for older adults. We have highlighted that the discharge planning process often overlooks the social and psychological needs of older and frail individuals whilst focusing on more traditional aspects of care, i.e., medication use and changes. Discharge from hospital should not only consistently account for physical capability but also incorporate bio-psycho-social assessments and plan for how psychologically vulnerable older adults can transition back into the community safely. We suggest the following key considerations when planning discharge for older adults in partnership with health and social care, including pharmacy:

- Older adults and their nominated contacts or informal caregivers should be equal partners in shared decision-making pertaining to their discharge.
- Ascertainment of psychological morbidity experienced by patients early on during hospital admission will inform how the immediate and longer-term management of patient issues such as loneliness, low mood, depression, and anxiety are incorporated into the discharge planning process.
- Healthcare professionals have a responsibility to ensure information provided has been understood by the patient and their formal and informal caregivers.
- Clear patient-centred communication amongst the multidisciplinary teams, including primary care/family physicians, will ensure patients receive relevant written information about their treatment and recovery timescales and their care needs, including any plans for rehabilitation or community-based activities. These vital pieces of information must be shared with and between service providers, professionals, older adults, and their formal/informal caregivers.

## 5. Conclusions

This systematic review highlights the importance of effective post-discharge planning for the older people, as well as the biopsychosocial requirements for older adults who are becoming an increasingly vulnerable section of the population as life expectancy increases. Our findings underscore the importance of comprehensive assessments spanning physical, social, and psychosocial domains, as well as enhanced communication between older adults, hospital-based multidisciplinary teams, and home care providers to improve the coordination of care. Planning discharge early during admission, with the involvement of the older person and their caregivers, is essential as it not only informs a better, holistic discharge process but also promotes recovery at home.

## Figures and Tables

**Table 1:** Inclusion and exclusion criteria.

Inclusion Criteria	Exclusion Criteria
Aged > 65 years	Aged < 65 *
Post-discharge	Pre-discharge
Psychological distress	Dementia/severe cognitive impairment
Loneliness	Palliative care
Anxiety	COVID-19
Depression	
Length of hospital stay	

* Studies should exclusively include participants/patients >65 years.

**Table 2:** Summary information of each study.

Author	Year of Publication	Setting	Country	Mean Age (Range)	Female
Greysen SR et al. [17]	2014	Urban teaching hospital	USA	63.0 (55–84)	33.0%
Andreasen J et al. [18]	2015	Hospitals	Denmark	80.6 (69–93)	50.0%
Lee DA et al. [21]	2017	Subacute or inpatient rehabilitation wards	Australia	78.4 (70–86)	58.0%
Brown A et al. [20]	2020	Hospitals and rehabilitation centres	Australia	78.4 (70–86)	57.0%
Olsson A et al. [22]	2020	Hospital	Sweden	84.0 (70–93)	60.0%
Martinsen et al. [19]	2015	Intermediate care (IC) unit	Denmark	79.0 (60–94)	75.0%
Reichardt et al. [23]	2019	Acute hospital environment	The Netherlands	79.6 (73–86)	49.0%

**Table 3:** Study characteristics and summary of findings.

Author(s)	Aim of the Study	Design and Sample	Data Analysis	Summary of Findings
Greysen et al. [17]	A study of vulnerable older adults describing their experience of recovery at home and need for a successful transition 30 days after discharge	Qualitative analyses of 24 participant interviews	Coded interviews using the constant comparative method. Categories extracted from the data	Participants reported functional limitations, difficulty with mobility and self-care, social isolation and challenges with poverty. Plan for post-discharge care had ‘missing pieces’
Andreasen J et al. [18]	Explore how frail older people experience daily life 1 week after discharge from an acute admission	Qualitative analyses of 14 participant interviews	Iterative Preliminary categories extracted from the data	Participants reported major stressors such as physical disability, loneliness, inactivity, and contact with healthcare systems Transition home experienced as unsafe
Lee DA et al. [21]	Temporal relationship between depressive symptoms, falls, and participation in physical activity post-discharge	Prospective cohort study of 311 participants surveyed based on Fiske’s behavioural model for 6 months	Logistic regression analyses	Falls positively associated with depressive symptoms Depressive symptoms negatively associated with physical activity levels Physical activity levels were negatively associated with falls
Brown A et al. [20]	Applied the Social Antecedent Model of Psychopathology (SAMP) to identify factors at discharge and future symptoms at 3- and 6-month post-discharge home	Prospective cohort study of 286 participants	Multiple regression analysis	Elevated anxiety, low levels of social support, and low perceived coping ability associated with depressive symptoms
Olsson A et al. [22]	Describe older adults’ experience of their life situation after hospital discharge	Qualitative analyses of 15 participant interviews	Audiotape recording. Categories extracted from the data	The main theme identified that participants longed to be independent again
Martinsen et al. [19]	Explore older peoples’ experiences of being back home after a stay in an intermediate care unit	Qualitative Phenomenological analyses of 12 participant interviews	Audiotape recording and phenomenological analysis	Transition home characterised by uncertainty Shock experienced on coming home, feeling dependent on others, a sense of social isolation and fear of losing functional ability
Reichardt et al. [23]	Investigates predictive value of depression using the Geriatric Depression Scale-15 (GDS-15) to predict outcome post-discharge	Prospective multicentre cohort study of 401 participants	Univariate and multiple logistic regression	Depression total score associated with increased mortality risk Hopelessness is a unique predictor of mortality Apathy is a common symptom in response to hospitalisation

## Data Availability

Further information can be obtained from the corresponding author.

## Appendix A

**Table A1:** Search terms used in Medline, PsycINFO, and CINAHL.

NB: S3 = S2 + S1/S5 = S3 + S4/S7 = S5 + S6/S12 = S8 + S9 + S11/S13 = S12 + S7 S1–S7 = free text subject heading S8–S11 = suggested subject headings S13 = free text subject heading + suggested subject headings	
Search ID	Search Terms
S1	old* OR elder OR geriatric OR aged
S2	Hospital* N3 discharge*
S4	stress OR wellbeing OR psychological OR loneliness OR “social* isolation*”
S6	house* OR home OR nursing home OR care home OR residential care OR rehabilitation OR community-living
S8	(“Aged, 80 and Over”) OR (MH “Frail Elderly”) OR (MH “Aged”)
S9	(“Patient Discharge”) OR (MH “Discharge Planning”)
S11	(“Anxiety Disorders”) OR (MH “Mental Health”) OR (MH “Psychology”) OR (MH “Health Behaviour”) OR (MH “Recreational Drug Use”) OR (MH “Smoking”) OR (MH “Mental Disorders”) OR (MH “Psychological Distress”) OR (MH “Cognitive Psychology”) OR (MH “Inhibition, Psychological”) OR (MH “Depressive Disorder”) OR (MH “Depression”)
